# Listening to the multidisciplinary care team: exploring the pediatric palliative care needs in advanced chronic kidney disease

**DOI:** 10.1007/s00467-025-06728-y

**Published:** 2025-02-27

**Authors:** Vanessa Nenner, Hendrik Napierala, Maria Agnes Jonas, Nina Kubiak, Julia Thumfart

**Affiliations:** 1 Department of Pediatric Gastroenterology, Nephrology and Metabolic Diseases, Charité Universitätsmedizin Berlin, Corporate Member of Freie Universität Berlin and Humboldt Universität zu Berlin, Augustenburger Platz 1, 13353 Berlin, Germany; 2 Institute of General Practice and Family Medicine, Charité Universitätsmedizin Berlin, Corporate Member of Freie Universität Berlin and Humboldt Universität zu Berlin, Berlin, Germany; 3 Department of Pediatric Respiratory Medicine, Immunology and Critical Care Medicine and Cystic Fibrosis Center, Charité Universitätsmedizin Berlin, Corporate Member of Freie Universität Berlin and Humboldt Universität zu Berlin, Berlin, Germany; 4 Clinic for Internal Medicine, Psychosomatics/Psychotherapy, Charité Universitätsmedizin Berlin, Corporate Member of Freie Universität Berlin and Humboldt Universität zu Berlin, Berlin, Germany

**Keywords:** Pediatrics, Advanced care planning, Supportive care, Conservative treatment, End-of-life care

## Abstract

**Background:**

Pediatric palliative care (PPC) aims to improve the quality of life for children with life-limiting conditions, such as advanced chronic kidney disease (CKD), from the time of diagnosis. However, PPC is not commonly integrated into routine pediatric nephrology care. This study explores the perspectives and experiences of healthcare providers (HCPs) to better understand the experiences and specific barriers related to PPC integration for children and adolescents with advanced CKD.

**Methods:**

We conducted a qualitative study with 23 HCPs, including nurses, psychologists, social workers, and physicians from seven German pediatric nephrology centers, analyzing semi-structured focus groups and individual interviews using structured content analysis.

**Results:**

Five main categories emerged from the analysis, revealing HCPs’ perceptions of CKD as a life-limiting condition, HCPs’ moral distress in addressing end-of-life issues, and barriers to PPC integration. Although HCPs reported comprehensive multidisciplinary support for end-of-life situations, a lack of interprofessional communication occasionally hindered coordinated care. HCPs rarely addressed CKD’s life-limiting nature proactively. A fear of diminishing hope led HCPs to avoid conversations about prognosis unless in response to a therapeutic crisis. PPC was mostly reserved for end-of-life cases, as HCPs associated PPC with terminal care and expressed concerns over distressing families.

**Conclusions:**

This study highlights the gap between guidelines recommending early integration of PPC and daily nephrology practice, which tends to introduce PPC late in the course of the disease. Training for nephrology teams could improve the quality of life for children with advanced CKD and their families by promoting early integration of primary PPC principles.

**Graphical abstract:**

**Figure:**
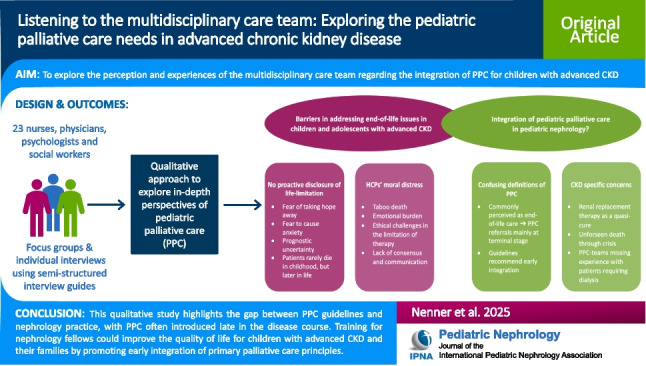
A higher resolution version of the Graphical abstract is available as Supplementary information

**Supplementary Information:**

The online version contains supplementary material available at 10.1007/s00467-025-06728-y.

## Introduction

Over the past three decades, pediatric palliative care (PPC) has evolved from a narrow focus on terminal hospice care to comprehensive care for all serious illnesses, beginning at diagnosis and continuing alongside disease-directed treatments [1]. Today, PPC is a right for all children with life-limiting or life-threatening diseases [2]. The World Health Organization defines palliative care as an approach that improves the quality of life of patients and their families who are facing the problems associated with life-threatening illness, through the prevention and relief of suffering by means of early identification and correct assessment and treatment of pain, and other problems, whether physical, psychosocial, or spiritual [3]. PPC encompasses both primary PPC, which is provided by primary healthcare providers (HCPs) to meet routine needs, and specialized PPC, which is usually provided by palliative care specialists [4]. Primary PPC consultations focus on managing basic symptoms and facilitating discussions about prognosis and treatment goals. In contrast, specialized PPC consultations focus on more complex symptoms, including refractory pain, profound psycho-existential distress, and support in cases of near futility.

In daily clinical routine care especially in pediatric subspecialties beyond oncology, the implementation of PPC is increasing. This is also demonstrated by the amount of published papers and the foundation of palliative care working groups. In the field of pediatric nephrology, for example, there is now an initiative by the International Society of Nephrology to define needs and implementation possibilities in a global approach.

The trigger for all these initiatives is the idea to optimize the care of our patients and support the whole family. This patient-centered care approach sees palliative and routine clinical care as concurrent care and not only as hospice or end-of-life care [5, 6].

Due to enormous medical progress, the life expectancy of children and adolescents with chronic kidney disease (CKD) has increased significantly over the last decades. However, the morbidity and mortality of affected individuals is still considerable, especially on dialysis [7]. Health-related quality of life (HRQOL) is significantly reduced compared to both healthy people and children and adolescents with other chronic diseases such as diabetes mellitus or cardiac disease [8, 9]. This impact on HRQOL may be even more pronounced in children with severe comorbidities besides CKD.

Thus, PPC is essential for children and adolescents with CKD, particularly in advanced stages (≥ stage 4), making routine primary PPC, and specialized PPC in select cases, recommended from diagnosis and continuing regardless of therapeutic goals [10]. However, in pediatric nephrology, integration of palliative care is lacking in the routine medical practice. We were able to show that HCPs who work in pediatric nephrology wish for more integration of PPC, e.g., through better training in palliative care [11]. As this study was only designed as a quantitative survey, we were unable to evaluate the exact needs and perception of the HCPs.

In the study presented here, a qualitative descriptive approach was used, in order to explore the perception and experiences of HCPs regarding the integration of PPC for children and adolescents with advanced CKD.

## Methods

### Study design

A qualitative descriptive approach was followed using focus groups and individual interviews based on interview guides. The study was approved by the ethics committee of the Charité Universitätsmedizin Berlin (EA2/234/17). To report the study, the Consolidated Criteria for Reporting Qualitative Research (COREQ), framework was used [12].

### Setting and participants

The participants were medical, psychosocial, and nursing professionals from seven of a total of 13 kidney centers for children and adolescents in Germany who offer dialysis and transplantation. The kidney centers were selected using purposive sampling, aiming to balance differences in center size and previous experience with PPC. For each kidney center, a local contact person identified members of the multidisciplinary pediatric nephrology team who were willing to participate in the study. They were invited by N.K. and V.N. by email with an information letter about the background of the study, the methods, and data protection. Recruitment of participants continued until data saturation was reached. In total, 28 HCPs were approached, but five did not respond and therefore did not participate in the study. All participants gave written informed consent. Participants’ sociodemographic data were collected using a pre-interview questionnaire. Variables included gender, age, profession, and years of work experience.

### Data collection

Based on the research team’s experience from previous studies, a semi-structured interview guide was devised for the focus group interviews in accordance with Helfferich’s recommendations (see Supplementary Materials) [11, 13, 14]. Its applicability was assessed in a multidisciplinary qualitative research workshop. Additionally, two clinical case vignettes were developed as prompts (see Supplementary Materials). In January and March 2024, two face-to-face focus groups were conducted at different nephrology centers, lasting 44 and 99 min, respectively. The focus groups were attended by at least one member of each professional discipline within the nephrology team, as well as a specialist in PPC. The participants received the case vignettes via email the day before. V.N., a master’s student of clinical psychology, was the moderator; J.V., a medical doctoral student, and N.K., a clinical psychologist in pediatric nephrology and a member of the research group, were co-moderators. They took notes and kept track of time.

A second semi-structured interview guide was developed for the individual interviews and tested in a pilot interview (see Supplementary Materials). Between January and August 2024, 11 interviews with HCPs who did not participate in the focus groups were conducted, either in person or online, and lasted between 26 and 55 min. J.V. and V.N. interviewed the participants and were not acquainted with them. These interviews did not use case vignettes, but an episodic approach centered on a critical case described by the HCPs.

### Data analysis

Interviews were audio-recorded and transcribed verbatim. Transcripts were not returned to the participants to avoid corrections for reasons of social desirability. Data were analyzed using a deductive-inductive approach based on Kuckartz’s structured content analysis and supported by MAXQDA© software (version 24) [15]. Initial data analysis was carried out by V.N. The coding system was revised by N.K. and some subcategories were merged. The results were discussed and reviewed by the entire research group.

## Results

### Participant characteristics

A total of 23 HCPs participated in this study: seven nurses, four psychologists, four social workers, and eight physicians (among which two nurses and one physician were specialized in the field of PPC) from seven kidney centers (Table 1). Most of the participants had more than 10 years of professional experience in pediatric nephrology.

**Table 1:** Characteristics of the participants (*N* = *23*)

	HCP focus groups (*n* = 12)	HCP interviews (*n* = 11)	Total, *N* = 23 (%)
Gender			
Female	9	7	16 (70)
Male	3	4	7 (30)
Age (years)			
< 25	0	0	0 (0)
25–35	3	1	4 (17)
36–45	2	1	3 (13)
46–55	4	5	9 (39)
> 55	3	4	7 (30)
Professions			
Physicians	5	3	8 (35)
Psychologist	2	2	4 (17)
Social worker	1	3	4 (17)
Nurses	4	3	7 (30)
Field of experience			
Pediatric nephrology	10	11^a^	21 (91)
Palliative care	2	0	2 (9)
Professional experience (years)			
< 2	1	0	1 (4)
2–5	1	2	3 (13)
6–10	1	1	2 (9)
> 10	9	8	17 (74)
Religious/spiritual			
Not at all	1	2	3 (13)
Not very	6	3	9 (39)
Somewhat	3	5	8 (35)
Very	2	1	3 (13)
Religion			
No	7	3	10 (43)
Protestant	5	3	8 (35)
Catholic	0	5	5 (22)

^a^One healthcare professional was also trained in palliative care

### Categories

Five main categories emerged from the interview transcripts, reflecting HCPs’ approaches to death and dying in pediatric nephrology and their attitudes towards the integrations of PPC: (1) status quo of current care; (2) CKD as a life-limiting condition; (3) dealing with death and dying; (4) HCPs’ moral distress; (5) PPC for advanced CKD. Table 2 provides an overview of the resulting categories and their subcategories.

**Table 2:** Overview of main categories and subcategories

Main categories	Subcategories
Status quo of current care	Multidisciplinary cooperation HCPs’ contentment Need for improvement
CKD as a life-limiting condition	No proactive disclosure, but honest answers Fear of taking away hope Fear to cause anxiety Prognostic uncertainty Most patients die in adulthood Life-limitation becomes important when …
Dealing with death and dying	Bad news Support for the whole family system Current support services Advanced care planning
HCPs’ moral distress	Taboo death Emotional burden Lack of consensus and communication Ethical challenges in limiting therapy Coping mechanisms
PPC for advanced CKD	Confusing definitions of PPC Practical experience with PPC CKD specific concerns Time of integration Training in PPC

*HCP* healthcare providers, *CKD* chronic kidney disease, *PPC* pediatric palliative care

Overall, HCPs perceived that care in the situation of death and dying was already adequately organized. They emphasized the multidisciplinary approach, as all disciplines would be needed to enable patients to die with dignity and to support each other in this challenging scenario. In the pediatric nephrology centers, affected families had access to a range of support services throughout the dying process, from the fulfillment of last wishes to spiritual care and accompaniment at the patient’s funeral.

However, further analysis will focus on the barriers and difficulties identified. A selection of categories is presented below to highlight key aspects of the analysis. Representative quotes for these categories are shown in Tables 3, 4, and 5.

**Table 3:** Category 1: CKD as a life-limiting condition

Quote	Quotation	Participant
1a	As a patient, I also expect my doctor to tell me if I have a chronic illness that is likely to shorten my life, because I naturally plan my life accordingly. Maybe you do an education that is a bit shorter if you know that the time is more valuable. And it’s exactly the same for parents, maybe they’ll think about a completely different path in life if they know that everything isn’t quite as safe and not quite as long-lasting as with a healthy child	Psych 1
1b	However, I often get the impression that the parents then withdraw their questions [about life-limitation] once the scenario has stabilized. Of course, that’s something that people tend to try to suppress	Phys 7
1c	And I wouldn’t address it [life expectancy] either, to be honest, because if someone only turns 50 instead of something like 78 or what, then for a child of eight or ten years or three years it doesn’t really matter yet, it doesn’t really matter yet	Soc 4
1d	And on the one hand, you don’t want to take away their hope and say that’s it. And even a new kidney is basically just a replacement therapy. You don’t want to tell them that. But it’s actually the case that things can go down again. (…) And I don’t know, I personally don’t always manage to say that it’s getting worse and worse. I always say, “Oh, it’ll be fine next time, we’ll check it again in two weeks.”	Nurs 7
1e	So, if I imagine the parents here, if we were to make this an issue, I think that the big issue would be hopelessness	Soc 2
1f	The most important signal is actually to give courage. The vast majority of patients we deal with can be told that yes, their child has a serious illness, no, we don’t want to sugarcoat it, but there is also hope that their child will grow old	Psych 4
1g	To be honest, this [life-limitation] is something that I don’t really notice now. The topic is always brought up when it comes to non-adherence or problems with adherence	Soc 3
1h	But it’s another form of life-limitation in oncology, I don’t know if that’s still the case, but it’s often decided whether the patient dies or not. For us it’s not really like that, this decision, it’s not really on the table, but there’s always something and something else and then dialysis, transplant, transplant is lost, dialysis again and it’s a bit like living in a circle	Phys 1
1i	So, we treat the children until they are 18, 21 at the most. Then they are gone, then they are transferred to the adult centers. (…) If they’ve been out of the center for a long time and then the case arises, that’s just this life-shortening thing that we know we might not even notice	Nurs 6
1j	We have a lot of children with serious chronic illnesses and they all have a limited life expectancy. And we also communicate this with the parents and many of them will only really start to have problems afterwards, when they are growing up in childhood and adolescence. This means that one way or another, we will eventually get rid of the problem here in pediatrics	Phys 6
1k	It’s a statistically large number, it’s calculated and then many people can’t really do anything with it. This is a person with a serious illness where there are many possible complications. And these complications can also lead to death at some point. And that’s why the shortened life expectancy is statistically there. But individuals can grow old. And that is what we actually convey to families	Psych 4
1l	But we hope that perhaps by the time the child is 30, we will have better therapies or other approaches to kidney replacement therapy. Of course, we hope that everything will be better in ten years’ time	Phys 7
1m	And then the question comes up from time to time and is then also honestly discussed that you simply carry a different cardiovascular risk with you due to this renal insufficiency that you have and that can be life-limiting. However, we also put it into perspective, or rather, yes, none of us can look into the crystal ball and no one knows whether they won’t develop a tumor and therefore die sooner than, rather than from renal insufficiency	Phys 8

*Psych* psychologist, *Phys* physician, *Nurs* nurse, *Soc* social worker

**Table 4:** Category 2: healthcare providers’ moral distress

Quote	Quotation	Participant
2a	Last year we had a young patient who was perhaps in her mid-20 s, who unfortunately didn’t recover properly after various operations. That really, really got to us all. We also questioned whether everything had really gone right and whether certain things should really have happened	Nurs 6
2b	To be honest, I’ve never had a single supervision session in my entire life	Nurs 7
2c	Physicians are socialized to life during their studies in such a way that death has no place there	Phys 3
2d	So even if it is now a palliative setting, it would be difficult for me now, if I were the physician treating her, to let her die of hyperkaliemia. (…) I would at least try to offer peritoneal dialysis. But in nephrology I always find that somehow, you can do something, even if you can’t transplant, you can somehow offer a substitute procedure	Phys 3
2e	I sometimes have the feeling that the subject of death is being given a very strict bow and that the word is not even mentioned and that it can come to an end. That you push it away SO much that the parents think okay, I’m getting a new kidney, my child is healthy, I’m done	Nurs 2
2f	Because there was a scenario where the other children on hemodialysis noticed that she was no longer there. And one of the nurses had also bought a children’s book on the subject of grief. And I wanted to take a look at it with another patient and talk to him about it. And what annoyed me a bit was that the mother had told him that she’d had a transplant and had gone home. Then it was more or less a done deal that I couldn’t talk to him about it anymore	Soc 3
2g	The physicians clearly said no, we will inform the local court, the child should live, against the will of the parents, yes, what is right? I can’t say, but it certainly poses a difficult problem	Phys 3
2h	More often, I would almost say when we have differing opinions in the team. Then we regularly have an ethics consultation meeting here, which, yes, in the end it’s not ethics that make a decision, but they mainly moderate it. This dissent, so that you can simply put it on the table openly and reflect on it	Phys 7
2i	When abuse happens in an institution, it’s called a traumatized system. And I also had the feeling that this, yes, partly hopeless, but also partly transplant-oriented scenario affected everyone and that it was very difficult to talk about it, to perhaps point out the ways and means in a conversation with the mother and also to show that it can also lead to death. I and my colleagues tended to follow the medical direction. And there was very little guidance or very little direction, shall I say	Soc 3

*Psych* psychologist, *Phys* physician, *Nurs* nurse, *Soc* social worker

**Table 5:** Category 3: pediatric palliative care for advanced CKD

Quote	Quotation	Participant
3a	Palliative care plays a background role in my day-to-day work, in that I know that if there are patients with a recognizably limited life expectancy, where treatments may be limited, that we would then think about palliative care, and then we would get in touch with our outpatient specialist palliative care team and would be in contact with them. I’ve just spoken to them recently to make sure that my perception corresponds to theirs. In recent years, they have had no patients with primarily nephrological diseases	Psych 4
3b	So I think that’s a bit of a first contact with palliative medicine for our center. There is a palliative care doctor who also has a team and who also comes from the oncology department and looks after the children in their area. But we haven’t had any other points of contact with her so far	Phys 2
3c	I only actually call in the palliative care team when things get complicated or when I need help with palliative care. At the moment when I only indicate palliative dialysis, the child is often still doing very well and that is very far from life-limiting. That’s why I don’t usually have them at the start because, to be honest, it’s a question of resources	Phys 8
3d	Yes, so [advanced care planning] is not standard, but also case-based. But yes, that’s part of this, so if it’s really palliative care, we can’t offer them anything else, I don’t do that with every dialysis patient that I dialyses palliatively	Phys 8
3e	There is a palliative care team, but I can’t think of any nephrological children where this has been used. It has been discussed before, but then it was also the case that the child wasn’t actually ill enough and the course developed positively and then no palliative team was deployed	Soc 3
3f	I got the palliative care team on board very early on or the bridge team with us, because I also wanted us to make sure that someone would visit regularly and see whether the parents were still coping or not. We did that for a year and a half until the parents said, well, it’s nice that they come round, but to be honest it doesn’t help us	Phys 8
3g	It’s more about these practical reliefs that are often missing, which leads to overloads, yes. But we also have families who then, right, outpatient, also palliative, yes, there are few families who make use of such support services, right. And go somewhere for a fortnight with the child. But those are actually the ones where the children are considerably more restricted, so more with complex disabilities	Psych 3
3h	Because I have to say, it [palliative care] is still connected in my head like that. So I know that it is also a lot of pain therapy and therefore of course improves quality of life, but for me the term palliative care is generally still very much associated with death	Nurs 2
3i	It [palliative care] doesn’t necessarily have anything to do with death. Of course it does, but that’s a much broader definition. (…) And when I listen to your statistics from Australia, they [children with advanced CKD] are basically all palliative. That would be my definition	Soc 2
3j	And I think that there are also some inhibitions about talking about the topic [palliative care]. So I would also say that I do have an threshold, because I always try to work in a trauma-sensitive way and not to prick somewhere where it hurts. So my fear here is simply that I will create or reinforce fears	Soc 3
3k	So when we say “palliative”, it’s about focusing on the fact that the child has a life-limiting illness. And at that moment, I tend to focus on death. Whereas if I provide psychosocial support and try to make things easier, like the steps that the family is facing, which we can already see as a team, until the transplantation takes place. And so that the quality of life is significantly improved again. Then we tend to look ahead. And try to accompany them to a positive point, so to speak	Psych 3
3l	Transplantation is the cure that is done. In this respect, dialysis in itself is not a life-limiting disease. There is a kidney replacement procedure that can be carried out for years. In this respect, this is not a classic case for palliation. (…) But it’s still a disease where the only treatment available is transplantation. Patients then have a decent life expectancy if everything goes well. But I wouldn’t involve the palliative care team in that, under any circumstances	Phys 6
3m	But I find it difficult when I think of our children who come to the outpatient clinic, they are often children who are not actually thinking about palliative care, but first of all about the shortest possible dialysis. Maybe even a transplantation via a living donation. And after that, the focus is on a positive quality of life and a great gain in quality of life, so to speak, and not on palliative care. (…) That doesn’t mean that I don’t mind, I just find it difficult to associate this large group of different patients with palliative care, yes	Psych 3
3n	But dying is also often a very acute thing. (…) Acute cardiac death, completely unpredictable. There is no starting point for outpatient palliative care. This is not a slowly progressing dying process. And that’s why it wouldn’t have been helpful for them to say, let’s start with palliative care now. That makes no sense at all	Psych 4
3o	So it’s also about what you can perhaps expect at the end of life with oncological diseases. I think it’s because they just have to be in a lot of pain. But I believe that this is more likely with children than with our nephrology patients, because if the nephrology patients are no longer treated properly, they will eventually develop uremia and then at some point—in double inverted commas—perhaps die of hyperkaliemia	Nurs 6
3p	We also have the palliative care team involved with some patients. But as I said, it’s sometimes difficult because they can perhaps bring in certain aspects, maybe a different perspective of course, which is sometimes helpful, but as I said, most of what they do is somehow already covered by our team anyway and in the past I’ve also thought, well, that wasn’t so helpful. Because the problem was that they come from the oncological field and don’t have that much experience with children on dialysis. There’s no need to talk about whether a patient who is still on peritoneal dialysis should be transferred to a pediatric hospice for the last three weeks	Soc 4
3q	Even in the first contacts with a family that has chronic renal insufficiency, a child with chronic renal insufficiency, to mention palliative care, I am absolutely opposed to that. It makes sense in very, very rare exceptional cases. In the vast majority of cases, it is important to encourage the family, not to gloss over anything, of course, but also not to tell them something false, that’s also clear, but to encourage them	Psych 4
3r	Because there are families where it [palliative care] is really necessary at such an early stage, where more complex malformations are present or the course of the disease has already begun intrauterine, so to speak, where the family has already gone through a certain path of suffering, perhaps it is already foreseeable that the child will have to stay in hospital for longer. That would really be very helpful. In the case of other patients who have been diagnosed in perfect health, but who are perhaps not yet so restricted in their everyday lives, I would perhaps find it a little difficult to say from the perspective that now comes a very dark time, we have to support you	Phys 7
3s	And if this takes place from the initial diagnosis, I actually think it’s good if there is continuous contact. I’m already aware that some children are getting worse as far as these stages of renal insufficiency are concerned. And then with a transplant, that also changes the situation again. My wish would be that this is then adapted to the situation, that it is simply individualized. But I can actually well imagine that this is something that can really provide quality through these different phases as a form of support	Soc 3
3t	So, I tend to think that further training is always good and can never hurt if someone has done additional training or perhaps specialized in something. Exactly. But I would think that this is/ and this further training is of course useful at the moment when the patient actually becomes palliative. But before that, I would think that it would be important to have more capacity to better accompany and support the families and also to keep a better eye on the siblings and to be able to offer even more	Psych 4
3u	And then there’s the lack of time and the comparison of several problems where you have the feeling that there are more urgent problems that need to be improved compared to palliative care. (…) So that’s, yes, there are huge problems at the moment, where we could really use three social workers to look after the children in this area, so that’s not at the top of my list of priorities	Phys 4
3v	If there was perhaps a bit more external support/ so if you could say that you have a palliative care team that is simply still there externally, which you could also integrate. Where you don’t have to say that we have to cancel doctors or nurses because we simply don’t have the resources	Phys 2

*Psych* psychologist, *Phys* physician, *Nurs* nurse, *Soc* social worker

### CKD as a life-limiting condition?

In daily practice, the life-limiting effects of advanced CKD often remain unaddressed, as there is mostly no proactive disclosure on this subject. Only a few HCPs noted that life limitation should be discussed proactively to allow patients and their families to plan their longer-term future (quote 1a). Patients and caregivers would ask about survival mainly in the context of transplantation or acute complications and would prefer to avoid the question of general life expectancy (quote 1b). At this young age, it would not matter to the affected families if the child lived to be “50 instead of 78” (quote 1c).

### Fear of taking away hope

However, when directly asked about life expectancy, the HCPs stated that they tried to give an honest answer but were not always capable of doing so (quote 1d). HCPs feared that educating patients and their caregivers about the life limitation could be too distressing and would promote hopelessness (quote 1e). Giving hope that the child is going to grow old was expressed as a priority (quote 1f). However, when addressing the consequences of medical non-adherence after transplantation, shortened life expectancy was used by HCPs (quote 1 g).

### Patients rarely die in childhood, but later in life

Unlike cancer, death from CKD is not typically imminent, as the condition does not progress in a linear fashion. Instead, it often involves a cyclical process of dialysis, transplantation, gradual graft loss, and a return to dialysis (quote 1 h). Thus, the pediatric HCPs reported little awareness of later critical incidents of their patients, as children and adolescents with advanced CKD usually die in adulthood (quote 1i). In pediatric nephrology, they “get rid of the problem” at some point (quote 1j).

### Prognostic uncertainty

According to the experience of the HCPs, the statistics of life expectancy would not necessarily reflect the individual patient, as patients before outlived poor prognosis (quote 1 k). Additionally, medical advances in the treatment of CKD were cited as a strong argument for optimism (quote 1 l). Thus, questions about statistical life expectancy should be relativized, as nobody “can look into the crystal ball” (quote 1 m).

### Healthcare professionals’ moral distress and death still as taboo

The death of a patient can constitute an emotional burden for HCPs (quote 2a). Team supervision was not always offered to cope with the emotional burden (quote 2b).

In medicine, one would be “socialized to life” (quote 2c). Hence, HCPs reported that even in a palliative setting, they had concerns to let patients die without at least offering dialysis as life-prolonging treatment (quote 2d). Communication about the possibility of death would sometimes be avoided, leaving parents with unrealistic hopes (quote 2e). In one case, the parents of a hemodialysis patient lied about the death of another patient on the dialysis ward, making it difficult for HCPs to discuss the passing of the child openly (quote 2f).

### Lack of consensus and communication

Decisions about treatment limitation would often raise difficult ethical issues for HCPs, especially when these decisions had to be made against the will of the caregivers (quote 2 g). In other cases, there were disagreements about the direction of treatment within the multidisciplinary HCP team, which is why ethical counselling was called upon to help resolve conflicts (quote 2 h). Interprofessional miscommunication was problematized. Physicians kept a mother in the hope of receiving a donor organ for her child, which in turn prevented the psychosocial team from preparing her for the child’s death (quote 2i).

### Established concepts of PPC in pediatric nephrology

While in some pediatric nephrology centers, PPC was seen as an integral part of pediatric nephrology, in others, PPC was seen more as a background option for critically ill patients at the end of life (quote 3a). There were also centers that had no prior contact with PPC at all (quote 3b).

HCPs did involve a specialized PPC team mainly as a last option in the occurrence of complications. Specialized PPC was integrated during intensive care unit stays or when additional expertise was needed in challenging cases of treatment limitation, palliative dialysis, or chronic pain (quote 3c). Similarly, advanced care planning was typically carried out only for dying patients (quote 3d). In fact, children died on the nephrology ward without the family being put in contact with a specialized PPC team because the child was “not actually ill enough” (quote 3e). In other cases, caregivers rejected the offer to integrate a specialized PPC team from the beginning or were later not satisfied with their services (3f). Outpatient PPC was in some centers used as a way of relieving the burden on families of children with complex disabilities, through short-term respite care in a children’s hospice or homecare at the end of life (quote 3 g).

### Confusing definitions of PPC

Among the HCPs, PPC was generally associated with end-of-life care, death, and dying (quote 3 h). Only a few HCPs were aware that PPC is today defined as supportive care from the point of diagnosis of a life-limiting illness (quote 3i). HCPs were concerned that the term “palliative” might cause anxiety or even trauma to patients and caregivers (quote 3j). While the psychosocial approach was perceived as positive, focusing on hope and resources, the palliative approach was perceived as negative, focusing on life limitation and death (quote 3 k).

### CKD-specific concerns for PPC integration

A common argument was that dialysis and transplantation today offer a reasonable life expectancy or even a quasi-cure, which precludes PPC (quote 3 l). HCPs found it difficult to associate their typical patient on dialysis or transplanted with PPC (quote 3 m). HCPs saw no indication for PPC, as death due to advanced CKD would usually happen suddenly through unforeseen therapeutic crises (quote 3n). The prevalence of pain at the end of life would also be lower with CKD than, for example, in cancer diseases (quote 3o). Moreover, HCPs experienced that specialized PPC teams were lacking experience with children on dialysis since they were mostly based in oncology (quote 3p).

### Time of PPC integration

Most HCPs dissuaded an early integration of primary PPC, preferring to focus on encouraging the family rather than confronting them with end-of-life issues (quote 3q). Conversely, some HCPs felt that early integration of PPC could be beneficial, particularly for families who have already experienced significant crises during the course of the illness (quote 3r). Some saw primary PPC as a way to improve the quality of care, if the time of integration was adapted to the family’s needs (quote 3 s).

### Future training in PPC

Most HCPs considered further training in primary PPC, e.g., enhancing communication skills, to be useful (quote 3t). However, training in PPC was not prioritized given other needs for improvement, such as the current shortage of qualified personnel (quote 3u). In the opinion of some HCPs, increased involvement of a specialized PPC team could improve care without placing an additional burden on the primary HCPs (quote 3v).

## Discussion

This qualitative study aimed to determine whether there is a need for PPC integration within pediatric nephrology from the perspectives of HCPs. Therefore, it was crucial to assess how advanced CKD is managed as a life-shortening and life-threatening condition and how PPC is established within pediatric nephrology.

Most importantly, HCPs were satisfied with the current care provided to dying patients and their families. However, a lack of interprofessional consensus or communication occasionally hindered coordinated care and conversations about impending death, leaving caregivers with unrealistic expectations. Although all participating kidney centers were equally resourced with inpatient and outpatient specialized PPC teams, the integration of primary PPC and specialized PPC services varied considerably across centers, depending on the practices and awareness of individual centers and their HCPs.

When kidney replacement therapy must be withdrawn, the integration of PPC for advanced CKD is clearly recommended [16]. According to today’s guidelines, PPC should be implemented already at the time of diagnosis of CKD [17]. Up to now, there are only a few studies investigating an early integration of PPC for the pediatric organ failure and transplant population [18–20]. There is a significant gap between guidelines and implementation in pediatric nephrology practice, as PPC is often provided late in the course of the disease and PPC resources are rarely used for children and adolescents with CKD [10]. This study illustrates that specialized PPC was integrated mostly at end-of-life scenarios. Occasionally, it was used as respite care for the families. The need for primary PPC was mostly negated by HCPs due to the availability of kidney replacement therapy as a life-prolonging treatment.

Integration of PPC is a matter of definition. Numerous studies, including the results of this study, show that PPC is mostly misunderstood as end-of-life care or hospice care, which causes reluctance to implement PPC and ultimately hinders early referral [6, 21]. Because of its negative association with death, alternative terms for PPC, such as “kidney supportive care,” are often preferred by patients and HCPs [22]. Also in this study, HCPs suggested using “psychosocial care.” While new terminology may ease fears, we chose to retain the term “palliative care” as it aligns with the World Health Organization’s broader definition, emphasizing comprehensive beyond end-of-life care. Evidence suggests that clear definitions of PPC can increase acceptance among HCPs [23]. However, we recognize that terminology can influence perceptions and acceptance, and this remains a topic open for discussion, particularly if adopting the term “kidney supportive care” could facilitate broader implementation of PPC for CKD. Ultimately, educating and engaging pediatric nephrology teams and affected families about the scope of palliative care, while remaining individually sensitive to language preferences, is essential.

In pediatrics, discussion of life-limiting or life-threatening prognosis is often limited as the focus is on promoting hope [24]. This was confirmed in our study. Life limitation of CKD was not proactively addressed. HCPs assumed that patients and their caregivers usually do not want to be informed about the subject and regarded it as too distressing.

In clinical practice, pediatric nephrology tends to prioritize management of CKD-related illnesses and optimization of dialysis but rarely addresses prognosis or treatment goals. Thus, the interviewees of this study indicated that prognostic conversations about life limitation in CKD were used more as a threat in case of non-adherence than as an opportunity to enable long-term planning for patients and their families. Instead, disclosure of prognosis and pediatric advance care planning has been shown to increase children’s and parents’ ability to anticipate decisions, support family coping, and improve their sense of control without undermining their hopes [24, 25].

In the interviews, a certain moral distress among the HCPs in dealing with the death of their patients was found, despite the fact that most of the HCPs were highly experienced, with more than 10 years of working experience in pediatric nephrology. Proactive integration of a specialized PPC team is shown to help reduce HCPs’ moral distress [26].

Finally, one of the main reasons for the reluctance of HCPs to integrate PPC is the fear that caregivers will feel they have given up on their child. In contrast, a recent survey showed that caregivers of dialysis-dependent children benefit from PPC consultations without feeling given up by the nephrology team [27].

Thus, the domains of PPC, such as psychosocial support, shared decision-making, and communication about prognosis and goals of care, should be proactively addressed in routine pediatric nephrology practice by the primary HCP team throughout the course of the disease, with the level of support tailored to the specific needs of the patient and their family. While most children and adolescents with advanced CKD do not usually require specialized PPC services [28], embedding primary PPC principles into routine pediatric nephrology practice would probably benefit all CKD patients. Pediatric nephrology teams, who are often more familiar with the affected families than specialized PPC teams, are well-positioned to provide such care, but the lack of PPC training in standard pediatric nephrology programs often leaves them lacking the confidence to perform primary PPC [29].

House and Wightman’s conceptual model for PPC integration in nephrology emphasizes that the multidisciplinary nephrology team should deliver tailored primary PPC at key moments, such as diagnosis, therapeutic crises, or transplantation [10]. The model proposes integrating PPC training into nephrology fellowship curricula to ensure the consistent application of primary PPC, even in settings with limited access to specialized PPC teams. This approach might be resource-efficient even in the face of financial constraints and staff shortages. Integrating such training for HCPs has been shown to increase referral rates to specialized PPC services for complex symptom management [30].

### Limitations

First, children with CKD and their families are key stakeholders in the needs assessment of PPC, but their perspectives were not included in this study. Second, the interest in the topic of PPC reported by the interviewed HCPs may represent a selection bias. The ones who are not interested in PPC might not have taken part in the study. Not all professional categories were represented equally across centers. While at least one doctor and one nurse were included from each center, only a total of four psychologists and social workers participated. This reflects the fact that not every kidney center in Germany has both a psychologist and a social worker. Finally, the interviewers were also part of the evaluation team. Nevertheless, investigator triangulation was ensured throughout the entire research process to guarantee the intersubjectivity of results.

## Conclusions

This qualitative study provides new insights into HCPs’ attitudes towards and practical experiences of PPC for children and adolescents with advanced CKD. While PPC is intended for all children with life-limiting or life-threatening conditions, including those with CKD, integration in pediatric nephrology is lacking. Primary PPC should be offered early alongside disease-directed treatment to address prognosis, enhance decision-making, and improve the quality of life for children with advanced CKD and their families. The majority of patients with less complex CKD may benefit from primary PPC during critical phases, such as initiation of dialysis or referral to transplantation, while some children may require specialized PPC due to their complex needs. Educating pediatric nephrology teams about the scope of palliative care is essential to promote broader implementation of PPC for children and adolescents with advanced CKD.

## Supplementary Information

Below is the link to the electronic supplementary material.

Graphical abstract (PPTX 347 KB)

Supplementary file2 (DOCX 29.2 KB)

Supplementary file3 (PDF 279 KB)

## Data Availability

All data which can be shared are in the article and the supplemental. Original interviews cannot be shared openly, to protect study participant privacy.
