# Supplementary material for: Listening to the multidisciplinary care team: exploring the pediatric palliative care needs in advanced chronic kidney disease
Source: Pediatr Nephrol. 2025 Feb 27;40(7):2341–51. doi: 10.1007/s00467-025-06728-y (PMC12116876; doi:10.1007/s00467-025-06728-y)
Supplement: Supplementary file 1 — Graphical abstract (PPTX 347 KB) [file 467_2025_6728_MOESM1_ESM.pptx]

## Slide 1
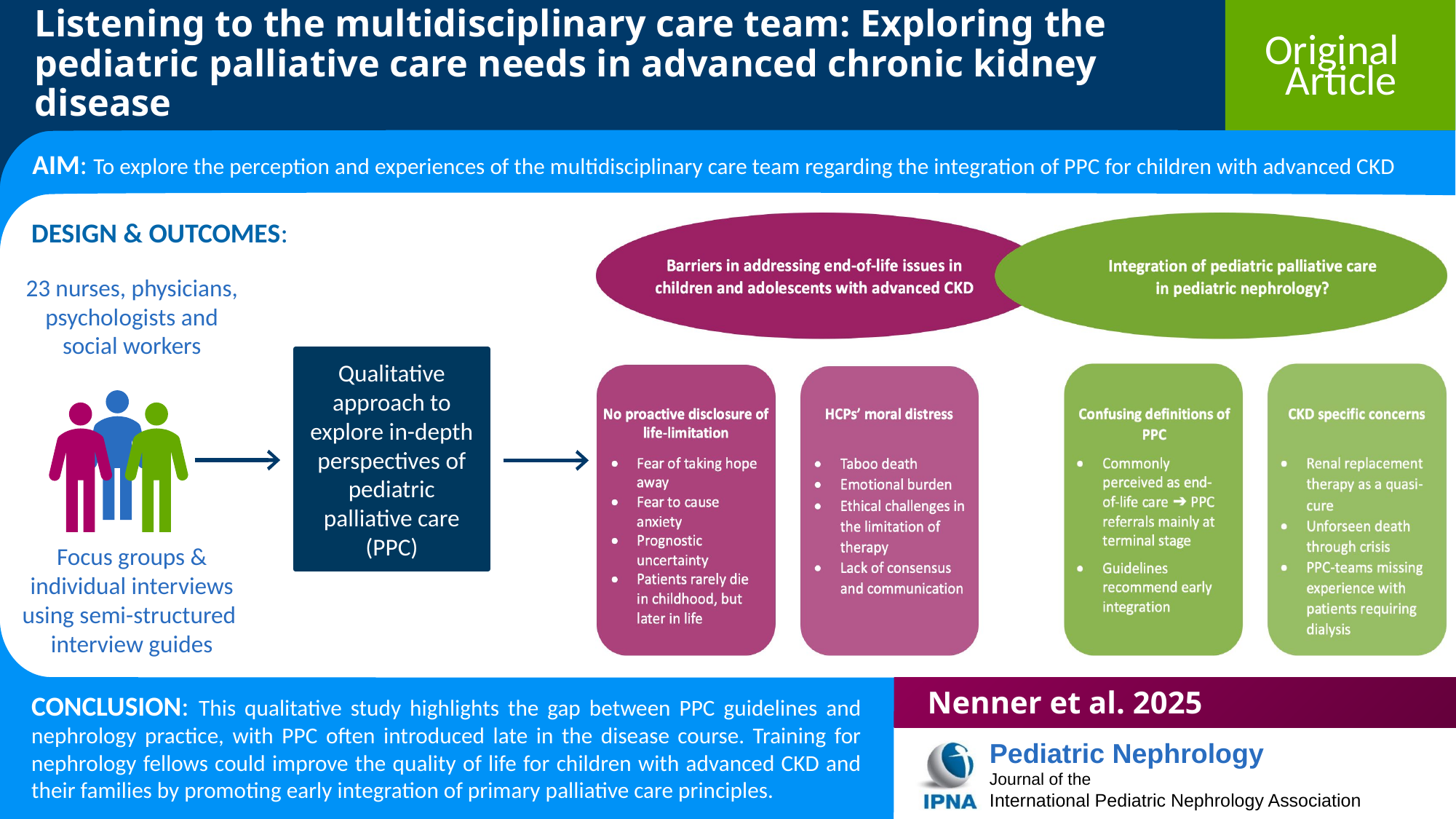

Listening to the multidisciplinary care team: Exploring the pediatric palliative care needs in advanced chronic kidney disease
AIM: To explore the perception and experiences of the multidisciplinary care team regarding the integration of PPC for children with advanced CKD
DESIGN & OUTCOMES:
23 nurses, physicians, psychologists and social workers
Qualitative approach to explore in-depth perspectives of pediatric palliative care (PPC)
Focus groups &
 individual interviews
using semi-structured
interview guides
Nenner et al. 2025
CONCLUSION: This qualitative study highlights the gap between PPC guidelines and nephrology practice, with PPC often introduced late in the disease course. Training for nephrology fellows could improve the quality of life for children with advanced CKD and their families by promoting early integration of primary palliative care principles.
