# Supplementary material for: Listening to the multidisciplinary care team: exploring the pediatric palliative care needs in advanced chronic kidney disease
Source: Pediatr Nephrol. 2025 Feb 27;40(7):2341–51. doi: 10.1007/s00467-025-06728-y (PMC12116876; doi:10.1007/s00467-025-06728-y)
Supplement: Supplementary file 2 — Supplementary file2 (DOCX 29.2 KB) [file 467_2025_6728_MOESM2_ESM.docx]

**Article Title:** Listening to the multidisciplinary care team: Exploring the pediatric palliative care needs in advanced chronic kidney disease

**Supplementary Table 1.** Interview Guide – HCP Focus Groups

| **Part I: Case vignettes** | | |
| --- | --- | --- |
| How would you proceed with regard to further treatment planning? | | |
| **Check** | **Specific questions** | **Maintenance** |
| - Only for case vignette 1: Reasonableness of *starting* renal replacement therapy - Patients' needs and quality of life - Communication with patient/family - Experience with specialized palliative care services | Have you ever been in a similar situation?   - If so, how did you and your team proceed at the time? - What went well? What went badly? - How did you involve the relatives?   How would you deal with a worsening of the condition during the course of the illness?   - What facilitates or hinders discussions about end-of-life treatment planning in everyday clinical practice?   Would you involve other practitioners?   - If so, which ones? | Non-verbal maintenance  Can you give an example?  What do you think?  Can you describe this in more detail?  What then? |

| **Part II: Concept of palliative care** | | |
| --- | --- | --- |
| What do you think about the term "palliative" in the context of pediatric nephrology? | | |
| **Check** | **Specific questions** | **Maintenance** |
| - Another term? | What do you understand by the concept of pediatric palliative care?  Should palliative care be routinely offered from the moment a life-limiting illness such as CKD is diagnosed?   - Why/why not? - If not, then when?   How would you imagine ideal (palliative) care in pediatric nephrology? | Non-verbal maintenance  What do you think?  Anything else?  Why (not)? |

**Supplementary Table 2.** Case Vignettes – HCP Focus Groups

|  | **Case vignette 1** | **Case vignette 2** |
| --- | --- | --- |
| **Patient** | 8-month-old boy | 16-year-old girl |
| **Diagnosis** | Galloway-Mowat syndrome | Renal dysplasia |
| **Forecast** | Progressive, severe developmental delay, steroid-resistant nephrotic syndrome, blindness, often death in preschool age | According to the US Renal Data System, the average life expectancy is 26 years (if always dialyzed) and 55 years (if always transplanted) |
| **Medical history** | Significant developmental delay (no head control, few spontaneous motor skills, severely impaired vision) | Pre-emptively transplanted at the age of 4 by means of a living donor from the mother, loss of the transplant at the age of 14 due to medication non-adherence |
| **Current status** | Rapidly progressing renal insufficiency due to steroid-resistant nephrotic syndrome with a current renal function of approx. 20% | 3 x week hemodialysis |
| **CKD stage according to KDIGO** | Stage 4  (kidney disease with severe impairment of kidney function) | Stage 5 D  (end-stage renal failure and dialysis) |
| **Family**  **situation** | 5 older siblings aged 3-13, behavioral and school problems with the eldest child, family has been looking for a larger rented flat for a long time (because of hygiene reasons), support from socio-pedagogic family help; Parents receive transfer payments | Only child  Parents working |
| **Occasion** | Further therapy planning with a special focus on the start of renal replacement therapy | Over the past few months, the patient has become increasingly resigned to her illness as she wants to avoid further organ loss in the event of another transplant. She comes across the word "mortality" while researching her illness on the internet and asks the dialysis nurse at the next connection how high the risk is that she will die earlier than her friends |
| **Additional**  **information** | Galloway-Mowat syndrome: A very rare multisystemic disease characterized by a neurodegenerative disorder | Pre-emptive: A pre-emptive kidney transplant is a kidney transplant that takes place before the patient has started dialysis, usually as a living donation |

**Supplementary Table 3.** Interview Guide – HCP Individual Interviews

| **Part I: Critical case description** | | |
| --- | --- | --- |
| Do you personally remember a particular case that dealt with the aspects of shortened life expectancy or dying? | | |
| **Check** | **Specific questions** | **Maintenance** |
| - Communication with patient and family | How did you and your team proceed with care planning and further treatment?  Were there any conflicts in the team?  What support was offered to the patient and their family by your center?  What went well? What didn't go so well? | Non-verbal maintenance  How did that go ...?  Can you tell me a bit more about this? |

| **Part II: Current care under the aspect of life-limitation** | | |
| --- | --- | --- |
| How do you perceive the life-limiting aspect of CKD in the care of children and adolescents? | | |
| **Check** | **Specific questions** | **Maintenance** |
| - Advanced Care Planning | Does life-limitation play a role in clinical care?   - Do you actively address the shortened life expectancy of children and adolescents with CKD as practitioners? - If yes: In which situations? - Have patients or relatives ever confided in you about a shortened life expectancy or other fears about the future?   What advantages/disadvantages do you see in honest and open communication about life expectancy? | Non-verbal maintenance  Can you tell us a bit more about this?  Why?  What else can you think of? |

| **Part III: Current care in the event of life-threatening crises and death** | | |
| --- | --- | --- |
| How do you deal with the situation in your center when the lives of children and adolescents with CKD are threatened by acute complications or imminent death is foreseeable? | | |
| **Check** | **Specific questions** | **Maintenance** |
| - Bio-psycho-socio-spiritual care - Patients' quality of life | What support services are available for the patient and their parents and siblings?  How is the topic of death and dying dealt within your team?  How is the topic of death and dying communicated with those affected and their relatives? | Non-verbal maintenance  What else can you think of? |

| **Part IV: Palliative care for children and adolescents with CKD** | | |
| --- | --- | --- |
| What do you know about pediatric palliative care? | | |
| **Check** | **Specific questions** | **Maintenance** |
| - Another term? - Specialized palliative care team? | What do you think of the term “palliative” in pediatric nephrology?  Is palliative care currently integrated into your routine care?   - If yes: In what way? At what point?   In your opinion, should palliative care for advanced CKD be routinely offered from the time of diagnosis?  What challenges do you see in integrating palliative care into the treatment of children and adolescents with CKD?  Could you imagine forming a specially trained team of staff from your center that would introduce primary palliative care into the routine care of CNI?   - If yes: Which professional groups should make up such a team? What further training content would be necessary? | Non-verbal maintenance  Why/why not?  And what about ...?  So you mean...?  Why/why not?  Can you give an example? |
